# Hypoglycemic episodes in hospitalized people with diabetes in Portugal: the HIPOS-WARD study

**DOI:** 10.1186/s40842-020-00114-3

**Published:** 2021-01-05

**Authors:** Sílvia Alão, João Conceição, Jorge Dores, Lèlita Santos, Francisco Araújo, Estevão Pape, Mónica Reis, Árcia Chipepo, Edite Nascimento, Ana Baptista, Vanessa Pires, Carlos Marques, Adriana De Sousa Lages, João Pelicano-Romano, Paula M. de Jesus

**Affiliations:** 1 MSD Portugal, R. Qta da Fonte 19, 2770-192 Paço de Arcos, Portugal; 2 MSD International GmbH (Singapore Branch), Medical Affairs, Singapore, Singapore; 3 Centro Hospitalar e Universitário do Porto, Porto, Portugal; 4 Centro Hospitalar e Universitário de Coimbra, Coimbra, Portugal; 5 Faculdade de Medicina da Universidade de Coimbra, Coimbra, Portugal; 6 Beatriz Ângelo Hospital, Loures, Portugal; 7 Hospital Garcia de Orta, Almada, Portugal; 8 Hospital de Vila Franca de Xira, Vila Franca de Xira, Portugal; 9 Centro Hospitalar Tondela Viseu, Viseu, Portugal; 10 Centro Hospitalar Universitário do Algarve – Faro, Faro, Portugal; 11 Centro Hospitalar de Trás-os-Montes e Alto Douro, Vila Real, Portugal; 12 Unidade Local de Saúde do Baixo Alentejo, Beja, Portugal

**Keywords:** Diabetes, Hypoglycemic episodes, Inpatients

## Abstract

**Background:**

We intended to estimate the proportion hypoglycemic/hyperglycemic emergency episodes in treated diabetes mellitus (DM) patients admitted to a hospital ward, and calculate the prevalence of risk factors for hypoglycemia and diabetic complications.

**Methods:**

In this cross-sectional, multicentered study, the observational data was collected by physicians from patient’s hospitalization to discharge/death. Statistical tests were 2-tailed considering 5% significance level.

**Results:**

There were 646 ward admissions due to hyperglycemic emergencies and 176 hypoglycemic episodes with a ratio hypoglycemia/hyperglycemia 0.27 for all DM patients. In T2DM patients the ratio was 0.38. These were mainly female (55.1%), functionally dependent (61.4%) and retired/disabled (73.1%). Median age was 75 years and median duration of disease 11 years. Half the patients were on insulin-based therapy and 30.1% on secretagogue-based therapy. Approximately 57% of patients needed occasional/full assistance to manage the disease. The most frequent risk factor for hypoglycemia was polypharmacy (85.0%). Hypoglycemia in the 12 months before admission was higher in insulin-based therapy patients (66.1%; *p* = 0.001).

**Conclusions:**

Hyperglycemic emergencies are the most frequent cause of hospitalization in Portugal, although severe hypoglycemic events represent a health and social problem in elderly/frail patients. There is still the need to optimize therapy in terms of the potential for hypoglycemia in this patient group and a review of anti-hyperglycemic agents to add on to insulin.

## Introduction

Diabetes mellitus (DM) is a chronic disease, characterized by hyperglycemia due to insulin deficiency, insulin resistance, or both [1, 2]. In Portugal the estimated prevalence of DM among adults (20–79 years) is 13.3% [3]. The relationship between the burden of hypoglycemia and hyperglycemia has been shifting and evolving [4, 5]. Achieving HbA1c targets < 7% (53 mmol/mol) has been shown to reduce microvascular complications of both type 1 and type 2 diabetes when instituted early in the course of disease [6]. International guidelines recommend lowering HbA1c to < 7.0% in most patients and to keep glycemic control well balanced and managed [7, 8]. However caution is needed in treating diabetes aggressively to near normal HbA1c goals in people with advanced age/frailty, with longstanding type 2 diabetes with or at significant risk of cardiovascular disease. Hypoglycemia is a limiting factor in achieving glycemic control and often related to poor clinical outcomes in patients with DM, making it difficult to manage [9–11]. Sometimes requiring assistance/hospitalization, hypoglycemia has a significant impact in patients’ lives. Typically, hyperglycemia related admissions (diabetes ketoacidosis (DKA), hyperglycemic hyperosmolar non-ketotic syndrome (HHNS)) are the major cause of DM related admissions in non-surgical wards. In the last decade there has been an increasing drive to improve glycemic target achievement with mean HbA1c at the primary care level dropping from 7.8% in 2010 to 6.8% in 2015 [3, 12]. While lowering HbA1c might be associated with an increase in the risk of hypoglycemia, Portugal has had a high uptake of innovative anti-hyperglycemic agents associated with lower risk of hypoglycemia (DPP4 inhibitors, SGLT2 inhibitors and GLP1 analogues) and a decrease in the use of sulphonylurea (SU) [3]. It is unclear if this trend impacted diabetes admissions.

Data from HIPOS-ER (HIPOS-Emergency Room) [13, 14] showed that 44.1% of patients with type 2 diabetes (T2DM) admitted to the emergency room with hypoglycemic episodes due to anti-hyperglycemic therapy were admitted to internal medicine/endocrinology wards, and discharged after 5 days on average. Results confirmed that hypoglycemia places a significant burden both to patient and society, accounting for adverse clinical and financial outcomes. In Portugal there is still a gap in the understanding of hypoglycemia admissions, hypoglycemia risk factors, and how they have evolved since the data from HIPOS-ER increased awareness of the magnitude of the problem.

This observational study aimed to evaluate the ratio of DM patients admitted in the ward due to hypoglycemia episodes vs. hyperglycemic emergencies, to calculate the prevalence of previously described hypoglycemia risk factors and diabetic complications in this population and to characterize patients by treatment group and type of diabetes.

## Materials and methods

The observational, cross-sectional, multicentered HIPOS-WARD (Hypoglycemia In Portugal Observational Study – WARD) study primarily assessed the proportion of hypoglycemic and hyperglycemia emergency episodes in DM treated adult patients that accounted for the admission to an endocrinology/internal medicine ward due to hypoglycemia. The proportion of hypoglycemia/hyperglycemia emergency episodes was also evaluated for T2DM patients.

Patients with diabetes and a hypoglycemic episode were characterized demographically and clinically (type and duration of diabetes, treatment group, diabetes complications, comorbidities, history of hypoglycemia, HbA1c, glycemia, repeated episodes during hospitalization). The prevalence of previously described hypoglycemia risk factors and diabetic complications were also calculated as well as hypoglycemia literacy of patients. Exploratory analyses by treatment group and type of diabetes were performed.

The study population included adult (≥18 years old) patients with diabetes treated with a known anti-hyperglycemic agent, hospitalized in the ward for a hypoglycemia event or as a result of a hypoglycemia episode in the non-hospitalized setting, in one of the 16 participating medical units (18 departments).

Hypoglycemia was defined according to the American Diabetes Association (ADA) [15, 16] definition of a modified Whipple’s triad (signs and symptoms suggestive of hypoglycemia that resolve with the administration of carbohydrates or glucagon and a glycemia < 70 mg/dL). Diagnosis was made by the attending ER physician, and ward admission was considered if: patients were admitted from the ER due to hypoglycemia; and/or patients that had an episode of hypoglycemia while at the hospital (outpatient clinic/other non-hospitalized setting) were admitted as a direct result of that hypoglycemia. Hospitalization was considered for patients admitted for at least 24 h for diagnosis/treatment.

Hospitalized hyperglycemic emergencies included DKA, HHNS and significant hyperglycemia. DKA and HHNS were defined according to ADA’s criteria [17]. Significant hyperglycemia was defined as glycemia ≥600 mg/dL or clinical symptoms (polyuria, polydipsia). Hyperglycemic emergencies were used to calculate the ratio hypoglycemia/hyperglycemia and not further assessed in this study.

Hypoglycemia literacy was evaluated by a non-validated questionnaire created and performed by the investigator to the patient/caregiver (if the patient was unable). Questions focused on the knowledge of the signs and symptoms of hypoglycemia and knowledge of strategies to prevent hypoglycemia.

Treatment subgroups were classified according to the anti-hyperglycemic agent therapy regimen: i) insulin-based therapy (included patients on insulin monotherapy/on insulin with or without other non-secretagogue drugs); ii) secretagogue-based regimen (included patients on secretagogue monotherapy (sulphonylurea and/or meglitinides), with/without other non-secretagogue drugs); iii) non-secretagogue-based therapy (included patients on drugs that do not include a secretagogue type drug (i.e. metformin, acarbose, glitazones, DPP4 inhibitors, GLP1 receptor agonists, SGLT2 inhibitors)); iv) combination of insulin and secretagogue (included patients that were treated with at least one insulin and one secretagogue type drug (other drugs may have been present)).

The total study duration was 21 months (November2016–August2018).

All study procedures were performed under standard clinical approach. Observational study data was collected by physicians from hospitalization admission to discharge or death. Data collected included sociodemographic, anthropometrics; diabetes characterization, risk factors; characterization of hypoglycemia episode; hypoglycemia literacy evaluation; laboratory and diagnostic exams; and discharge information.

The study was approved by the Hospital’s or local Ethics Committees and by the National Committee for Data Protection. Written informed consent was obtained from all participants.

### Statistical methods

Data were summarized using descriptive statistics (mean, standard deviation, median, minimum and maximum for quantitative variables; counts and percentages for qualitative variables). Missing values were not replaced. Percentages were calculated based on non-missing values.

The ratio of ward admissions due to hypoglycemia per hyperglycemic emergencies was estimated with corresponding 95% confidence interval (CI). The comparisons of anti-hyperglycemic agent therapy classes or type of diabetes were performed with Chi-square test, Fisher’s exact test or Monte Carlo methods (if assumptions were not met) for categorical variables. Quantitative variables were compared using ANOVA or Mann-Whitney test and Kruskal-Wallis test, if assumptions were not met.

Statistical tests were 2-tailed considering 5% significance level. Statistical analyses were performed using SAS® version 9.4 software (SAS Institute Inc., Cary, NC, USA).

## Results

### Ratio hypoglycemia/hyperglycemia

A total of 646 ward admissions were registered due to hyperglycemic emergencies (328 DKA, 203 HHNS, 115 significant hyperglycemias) in all patients with diabetes. In the same period, there were 176 ward admissions due to hypoglycemia episodes in DM patients. The ratio hypoglycemia/hyperglycemia was 0.27 (95%CI = 0.23;0.32) for all DM patients. Considering T2DM patients, there were 404 hyperglycemic emergencies and 152 hypoglycemia episodes, in a ratio of 0.38 (95%CI = 0.31;0.45).

### Baseline characterization

The 176 ward admissions due to hypoglycemia episodes are the focus of this analysis. Half the patients included in the study were on insulin-based therapy (50.0%), 30.1% were on secretagogue-based therapy, 9.7% on non-secretagogue therapy and 10.2% on insulin+secretagogue therapy. The sample was mainly female (55.1%) with median age of 75 years and mean body mass index (BMI) 26.0 kg/m^2^. A statistically significant association was found between treatment groups and median age (*p* = 0.009, insulin+secretagogue and secretagogue patients with higher values) and with BMI (*p* = 0.032, secretagogue-based patients had higher BMI). Approximately 19% of patients were institutionalized and 23% of the non-institutionalized were living alone. Most of the patients (73.1%) were retired/disabled and only 9.2% were working full or part time. The hypoglycemia episodes occurred mainly in T2DM patients (86.4%), with a median duration of disease of 11 years. More than half of patients needed occasional (27.8%) or full (29.5%) assistance to manage the disease, 61.4% were functionally dependent. The proportion of patients followed in primary health care was statistically higher (*p* < 0.001) in patients with secretagogue-based therapy (94.3%), compared to the other groups of treatment. Patients on insulin+secretagogue and insulin-based therapy had a higher median disease duration (*p* < 0.001). Half of the sample suffered from chronic diabetes complications related with the eye (50.0%: mostly retinopathy), atherosclerotic disease (47.8%: mostly peripheral artery disease) or nephropathy (47.4%: mean creatinine: 2.58 mg/dl; mean eGFR: 41.15 ml/min/1.73m^2^; mean eGFR basal: 47.60 ml/min/1.73m^2^; mean albumin: 248.42 μg/mg; median proteinuria: 344.00 mg). Presence of eye complications was higher in patients on insulin-based therapy (62.3%; *p* = 0.011). Other complications were not statistically different between treatment groups. HbA1c (*p* < 0.001) and the lowest glycemia (*p* = 0.041) in the ward showed statistically significant differences between groups: insulin-based patients had highest median HbA1c (7.6%) and insulin+secretagogue patients the highest median lowest glycemia (91.5 mg/dL). Approximately 48% of the patients had a hypoglycemia in the previous 12 months (mean 2.2 events/patient required assistance from other person; mean 0.5 events/patient pre-hospital assistance; mean 0.7 events/patient ER assistance). From those, 70.4% had hypoglycemia in the previous 30 days. One episode occurred while the patient was driving. The proportion of patients with hypoglycemia in the 12 months previous to admission was higher in patients on insulin-based therapy (66.1%) with statistically significant differences (*p* = 0.001) (Table 1).

**Table 1:** Baseline patient’s characteristics, overall and by class of anti-hyperglycemic agent

	Total (***n*** = 176)	Class of anti-hyperglycemic agent therapy				***P***-value
		Insulin-based therapy (***n*** = 88)	Secretagogue-based therapy (***n*** = 53)	Non-secretagogue-based therapy (***n*** = 17)	Insulin + secretagogue therapy (***n*** = 18)	
**Gender (female), n(%)**	97 (55.1)	42 (47.7)	33 (62.3)	11 (64.7)	11 (61.1)	0.269^a^
**Age, median (min;max), (years)**	75.0 (28;98)	73.5 (28;92)	78.0 (50;96)	72.0 (45;98)	78.5 (58;88)	**0.009**^**b**^
**BMI, mean (SD), (kg/m**^**2**^**)**	26.0 (4.6)	25.2 (4.7)	27.4 (4.3)	25.8 (4.7)	25.5 (4.6)	**0.032**^**b**^
**Patient institutionalized, n(%)**	33 (18.8)	13 (14.8)	11 (20.8)	2 (11.8)	7 (38.9)	0.122^c^
**Patient lives alone, n(%)**	33 (23.1)	19 (25.3)	8 (19.0)	4 (26.7)	2 (18.2)	0.880^c^
**Work status, n(%)**						**0.015**^**c**^
Full time	15 (8.6)	14 (16.1)	0 (0.0)	1 (5.9)	0 (0.0)	
Partial time	1 (0.6)	0 (0.0)	1 (1.9)	0 (0.0)	0 (0.0)	
Retired/disabled	128 (73.1)	55 (63.2)	46 (86.8)	11 (64.7)	16 (88.9)	
Other	31 (17.8)	18 (20.6)	6 (11.3)	5 (29.4)	2 (11.1)	
**Functionally dependent, n(%)**	70 (61.4)	33 (70.2%)	24 (60.0)	6 (60.0)	7 (41.2)	0.211^a^
**Type of diabetes, n(%)**						**< 0.001**^**c**^
Type 1	18 (10.2)	18 (20.5)	0 (0.0)	0 (0.0)	0 (0.0)	
Type 2	152 (86.4)	64 (72.7)	53 (100.0)	17 (100.0)	18 (100.0)	
Other form of diabetes	6 (3.4)	6 (6.8)	0 (0.0)	0 (0.0)	0 (0.0)	
**Diabetes duration, median (min;max), (years)**	11.0 (0.0;52.0)	17.0 (0.7;52.0)	9.0 (0.0;47.0)	1.0 (1.0;30.0)	18.0 (1.0;45.0)	**< 0.001**^**b**^
**Diabetes management, n(%)**						0.383^a^
Solely managed by self	75 (42.6)	36 (40.9)	23 (43.4)	10 (58.8)	6 (33.3)	
Solely managed by other	52 (29.5)	22 (25.0)	19 (35.8)	4 (23.5)	7 (38.9)	
Occasional assistance	49 (27.8)	30 (34.1)	11 (20.8)	3 (17.6)	5 (27.8)	
**Usual diabetes outpatient care, n(%)**^**d**^						**< 0.001**^**c**^
Primary healthcare	111 (63.1)	40 (45.5)	50 (94.3)	10 (58.8)	11 (61.1)	
Hospital outpatient	45 (25.6)	35 (39.8)	0 (0.0)	5 (29.4)	5 (27.8)	
Private consultation	7 (4.0)	5 (5.7)	1 (1.9)	0 (0.0)	1 (5.6)	
Other	11 (6.3)	8 (9.1)	0 (0.0)	2 (11.8)	1 (5.6)	
Without follow-up	2 (1.1)	0 (0.0)	2 (3.8)	0 (0.0)	0 (0.0)	
**Chronic diabetes complications, n(%)**^**d**^						
Eye complications	63 (50.0)	43 (62.3)	12 (40.0)	3 (20.0)	5 (41.7)	**0.011**^**a**^
Neuropathy	29 (26.1)	17 (27.9)	6 (21.4)	1 (9.1)	5 (45.5)	0.249^c^
Nephropathy	74 (47.4)	37 (48.1)	25 (53.2)	5 (31.3)	7 (43.8)	0.493^a^
Lower limb complications	24 (15.9)	16 (20.8)	4 (8.9)	2 (13.3)	2 (14.3)	0.393^c^
Known atherosclerotic disease	66 (47.8)	30 (44.8)	19 (46.3)	7 (46.7)	10 (66.7)	0.488^a^
Coronary heart disease	32 (57.1)	17 (63.0)	8 (53.3)	3 (50.0)	4 (50.0)	0.848^c^
Cerebrovascular disease	31 (55.4)	15 (57.7)	12 (70.6)	3 (50.0)	1 (14.3)	0.092^c^
Motor complications	19 (33.3)	11 (42.3)	6 (33.3)	1 (16.7)	1 (14.3)	0.462^c^
Peripheral artery disease	25 (61.0)	10 (58.8)	6 (54.5)	3 (50.0)	6 (85.7)	0.535^c^
Other atherosclerotic dis	8 (21.1)	4 (23.5)	1 (9.1)	1 (20.0)	2 (40.0)	0.612^c^
**Previous hypoglycemia in last 12 months, n(%)**	58 (47.5)	39 (66.1)	11 (28.9)	4 (30.8)	4 (33.3)	**0.001**^**a**^

*SD* Standard deviation

^a^ Chi-square test; ^b^ Kruskal-Wallis test; ^c^ Monte Carlo methods; ^d^ Each patient can have more than one

### Hypoglycemia episode characterization

The most frequent triggering causes of hypoglycemia were carbohydrate deficit (including ‘missed or delayed meal’ and ‘meal inappropriately low in carbohydrates’) (54.0%), acute illness (40.2%) and related to insulin therapy (34.3%). As determined by the investigators, approximately 15% of patients had associated complications as a result of hypoglycemia, most frequently neurologic and infectious complications (3 aspiration pneumonias, 2 lower urinary tract infections, 1 pyelonephritis, 1 sepsis and 1 conjunctivitis).

The median lowest glycemia recorded at ER admission or pre-hospital was 40.0 mg/dL (9.0–298.0 mg/dL) and 74.0 mg/dL (20.0–268.0 mg/dL) in the ward. A total of 63 patients (36.0%) had a repeated hypoglycemia during hospitalization, none of which led to further complications. The relative frequency of patients with hypoglycemia during the hospitalization was 47.1% on insulin-based patients, 26.4% on secretagogue-based patients, 29.4% on non-secretagogue and 16.7% on insulin+secretagogue patients (*p* = 0.018). There were no statistical differences in the length of stay or hospitalization outcome, however there were more deaths in the insulin-based patients.

The risk factors for hypoglycemia and the risk factors for complications following an event were also assessed. The most frequent risk factor was polypharmacy (> 5 distinct drugs), in 85.0% of patients, followed by age > 75 years (49.4%). Age > 75 years was statistically different between treatment groups with 72.2% patients from insulin+secretagogue and 35.3% patients from non-secretagogue regimen (*p* = 0.017). Heart failure was the most frequently reported (28.9%) risk factor for complications after an event (Table 2).

**Table 2:** Hypoglycemia episode characterization, overall and by class of anti-hyperglycemic agent

	Total (***n*** = 176)	Class of anti-hyperglycemic agent therapy				***P***-value
		Insulin-based therapy (***n*** = 88)	Secretagogue-based therapy (***n*** = 53)	Non-secretagogue-based therapy (***n*** = 17)	Insulin + secretagogue therapy (***n*** = 18)	
**Hypoglycemia episode, n(%)**	176 (100.0)	88 (50.0)	53 (30.1)	17 (9.7)	18 (10.2)	
**HbA1c, median (min;max)(%)**	6.9 (4.0;27.0)	7.6 (4.6;27.0)	5.8 (4.0;14.2)	5.8 (4.6;9.9)	7.0 (5.4;10.3)	**< 0.001**^**b**^
**Lowest glycemia, median (min;max)(mg/dL)**	74.0 (20.0;268.0)	56.0 (20.0;268.0)	80.0 (30.0;159.0)	76.5 (39.0;170.0)	91.5 (25.0;160.0)	**0.041**^**b**^
**Complications as result of hypo, n(%)**	26 (14.9)	15 (17.2)	7 (13.2)	0 (0.0)	4 (22.2)	0.240^c^
Major trauma	3 (11.5)	3 (20.0)	0 (0.0)	0 (0.0)	0 (0.0)	0.719^c^
Acute atherosclerotic event	1 (3.8)	1 (6.7)	0 (0.0)	0 (0.0)	0 (0.0)	> 0.999^c^
Other CV event	4 (15.4)	1 (6.7)	1 (14.3)	0 (0.0)	2 (50.0)	0.137^c^
Neurologic	6 (23.1)	6 (40.0)	0 (0.0)	0 (0.0)	0 (0.0)	0.089^c^
Infection	6 (23.1)	4 (26.7)	1 (14.3)	0 (0.0)	1 (25.0)	> 0.999^c^
Other	9 (34.6)	3 (20.0)	5 (71.4)	0 (0.0)	1 (25.0)	0.055^c^
**Repeated hypo during hospitalization, n(%)**	63 (36.0)	41 (47.1)	14 (26.4)	5 (29.4)	3 (16.7)	**0.018**^**a**^
**Lenght of stay in ward, median (min;max)(h)**	146.6 (1.6;1782.3)	159.6 (1.6;1782.3)	118.4 (19.4;781.9)	168.6 (34.7;935.0)	165.7 (34.4;1583.7)	0.275^b^
**Discharge destination, n(%)**						0.198^c^
Home	113 (64.2)	52 (59.1)	40 (75.5)	11 (64.7)	10 (55.6)	
Ambulatory	24 (13.6)	17 (19.3)	2 (3.8)	2 (11.8)	3 (16.7)	
Institution	30 (17.0)	12 (13.6)	10 (18.9)	3 (17.6)	5 (27.8)	
Other hospital	1 (0.6)	1 (1.1)	0 (0.0)	0 (0.0)	0 (0.0)	
Death	8 (4.5)	6 (6.8)	1 (1.9)	1 (5.9)	0 (0.0)	
**Risk factors for hypoglycemia, n(%)**^**e**^						
> 75 years	87 (49.4)	36 (40.9)	32 (60.4)	6 (35.3)	13 (72.2)	**0.017**^**a**^
Dementia	28 (16.7)	11 (13.4)	11 (21.2)	2 (12.5)	4 (22.2)	0.543^c^
Major sensorial impairment						
Blindness	13 (7.4)	9 (10.3)	2 (3.8)	0 (0.0)	2 (11.1)	0.297^c^
Deafness	6 (3.4)	2 (2.3)	1 (1.9)	0 (0.0)	3 (16.7)	0.050^c^
Hypothyroidism	14 (10.1)	9 (13.4)	3 (7.3)	1 (7.1)	1 (6.3)	0.811^c^
Adrenal failure	3 (2.2)	2 (2.9)	0 (0.0)	1 (7.1)	0 (0.0)	0.267^c^
Severe renal failure	19 (38.0)	11 (39.3)	4 (25.0)	1 (50.0)	3 (75.0)	0.263^c^
Hepatic failure	7 (4.1)	5 (5.9)	2 (3.8)	0 (0.0)	0 (0.0)	0.875^c^
Cachexia/significant malnourishment	19 (10.9)	15 (17.2)	2 (3.8)	1 (6.3)	1 (5.6)	0.068^d^
Brittle diabetes	21 (14.9)	15 (23.1)	4 (9.3)	2 (12.5)	0 (0.0)	**0.049**^**c**^
Weaning of corticosteroid drugs	10 (5.8)	4 (4.7)	1 (1.9)	4 (23.5)	1 (5.6)	**0.025**^**c**^
Previous hypo in last 12 months	58 (47.5)	39 (66.1)	11 (28.9)	4 (30.8)	4 (33.3)	**0.001**^**a**^
Lifestyle factors						
Irregular meals	52 (37.1)	28 (36.8)	13 (32.5)	6 (54.5)	5 (38.5)	0.606^c^
Shift work	4 (2.4)	4 (4.7)	0 (0.0)	0 (0.0)	0 (0.0)	0.515^c^
Other	7 (4.0)	5 (5.7)	2 (3.8)	0 (0.0)	0 (0.0)	0.872^c^
Polypharmacy	147 (85.0)	69 (81.2)	45 (84.9)	16 (94.1)	17 (94.4)	0.412^c^
**High risk for complications after the event**						
Coronary heart disease	32 (25.0)	17 (26.6)	8 (21.6)	3 (21.4)	4 (30.8)	0.897^c^
Cerebrovascular disease	31 (24.2)	15 (23.8)	12 (30.8)	3 (21.4)	1 (8.3)	0.613^c^
Heart failure	48 (28.9)	20 (23.8)	19 (36.5)	3 (23.1)	6 (35.3)	0.372^d^
Peripheral artery disease	25 (22.1)	10 (18.5)	6 (18.2)	3 (21.4)	6 (50.0)	0.151^c^
Osteoporosis	8 (7.6)	3 (5.4)	3 (10.0)	1 (9.1)	1 (12.5)	0.518^c^
Living alone	33 (18.8)	19 (21.6)	8 (15.1)	4 (23.5)	2 (11.1)	0.642^c^

^a^ Chi-square test; ^b^ Kruskal-Wallis test; ^c^ Monte Carlo methods; ^d^ Fisher’s exact test; ^e^ Each patient can have more than one; Polyphramacy: > 5 distinct drugs, for one or more conditions

The median time spent by patients in the ER was approximately 9.1 h and 146.6 h/6.1 days in the ward. Approximately 64% of patients were discharged back home, while 17.6% went to an institution/another hospital and 4.5% (8 patients) died (Table 2). About 43% of the patients were referred to hospital diabetes/endocrinology outpatient clinic and 32.9% to the general practitioner.

### Hypoglycemia literacy

Patients were evaluated on their knowledge of identifying, treating and preventing a hypoglycemia event (hypoglycemia literacy questionnaire). The questionnaire was answered by 77.1% (*n* = 135) of patients and 22.9% (*n* = 40) of caregivers. Approximately 75% (*n* = 132) of the patients knew ‘what is a hypoglycemia, ‘hypo’ or ‘a drop in blood sugar’. From these, 90.9% (*n* = 120) knew ‘how to identify it’, 84.8% (*n* = 112) knew ‘what to do in case of hypoglycemia’ (98.2% (*n* = 110) answered ‘ingest a form of simple carbohydrate’) and 63.6% (*n* = 84) knew ‘what to do to prevent it’.

### Subgroup analyses by type of diabetes

In the study population, 86.4% of patients had T2DM, 10.2% T1DM and 3.4% had another form of diabetes (3 patients ‘post pancreatectomy’, 1 patient ‘after surgery’, 1 patient ‘secondary’ and 1 patient ‘secondary to pancreatitis’). Due to its small size, the group ‘other type of diabetes’ was excluded from the inferential analysis. Characterization by type of diabetes is described on Table 3.

**Table 3:** Characterization by type of diabetes

	Total (***n*** = 176)	Type of diabetes		***P***-value
		Type 1 (***n*** = 18)	Type 2 (***n*** = 152)	
**Hypoglycemia episode, n(%)**	176 (100.0)	18 (10.2)	152 (86.4)	
**Gender (female), n(%)**	97 (55.1)	6 (33.3)	88 (57.9)	**0.048**^**a**^
**Age, median (min;max), (years)**	75.0 (28;98)	49.5 (28.0;74.0)	78.0 (35.0;98.0)	**< 0.001**^**b**^
**BMI, median (min;max), (kg/m**^**2**^**)**	26.0 (4.6)	22.9 (18.4;31.6)	26.3 (15.6;38.8)	**0.009**^**d**^
**Diabetes duration, median (min;max), (years)**	11.0 (0.0;52.0)	22.0 (4.0;52.0)	10.0 (0.0;51.0)	**0.001**^**b**^
**Diabetes management, n(%)**				**0.013**^**a**^
Solely managed by self	75 (42.6)	13 (72.2)	58 (38.2)	
Solely managed by other	52 (29.5)	1 (5.6)	50 (32.9)	
Occasional assistance	49 (27.8)	4 (22.2)	44 (28.9)	
**Usual diabetes outpatient care, n(%)**^**f**^				**0.001**^**c**^
Primary health care	111 (63.1)	4 (22.2)	104 (68.4)	
Hospital outpatient	45 (25.6)	11 (61.1)	31 (20.4)	
Private consultation	7 (4.0)	1 (5.6)	6 (3.9)	
Other	11 (6.3)	2 (11.1)	9 (5.9)	
Without follow-up	2 (1.1)	0 (0.0)	2 (1.3)	
**Chronic diabetes complications, n(%)**^**f**^				
Eye complications	63 (50.0)	13 (81.3)	48 (46.2)	**0.014**^**e**^
Neuropathy	29 (26.1)	7 (50.0)	19 (20.9)	**0.040**^**e**^
Nephropathy	74 (47.4)	6 (37.5)	67 (49.6)	0.359^a^
Lower limb complications	24 (15.9)	2 (11.8)	20 (15.6)	> 0.999^e^
Known atherosclerotic disease	66 (47.8)	2 (14.3)	62 (51.7)	**0.008**^**a**^
Coronary heart disease	32 (57.1)	2 (100.0)	29 (55.8)	
Cerebrovascular disease	31 (55.4)	1 (100.0)	30 (56.6)	
Motor complications	19 (33.3)	0 (0.0)	19 (35.8)	
Peripheral artery disease	25 (61.0)	1 (100.0)	24 (63.2)	
Other atherosclerotic dis	8 (21.1)	0 (0.0)	7 (20.0)	
**Previous hypoglycemia in last 12 months, n(%)**	58 (47.5)	12 (85.7%)	42 (40.4%)	**0.001**^**a**^
**Repeated hypo during hospitalization, n(%)**	63 (36.0)	7 (41.2%)	51 (33.6%)	0.530^a^
**Length of stay in ward, median (min;max)(h)**	146.6 (1.6;1782.3)			
**Discharge destination, n(%)**				0.052^c^
Home	113 (64.2)	13 (72.2)	98 (64.5)	
Ambulatory	24 (13.6)	5 (27.8)	16 (10.5)	
Institution	30 (17.0)	0 (0.0)	29 (19.1)	
Other hospital	1 (0.6)	0 (0.0)	1 (0.7)	
Death	8 (4.5)	0 (0.0)	8 (5.3)	
**Risk factors for hypoglycemia, n(%)**^**f**^				
> 75 years	87 (49.4)	0 (0.0)	86 (56.6)	**< 0.001**^**a**^
Dementia	28 (16.7)	0 (0.0)	28 (19.0)	0.081^e^
Major sensorial impairment				
Blindness	13 (7.4)	4 (23.5)	8 (5.3)	**0.021**^**e**^
Deafness	6 (3.4)	1 (5.9)	5 (3.3)	0.476^e^
Hypothyroidism	14 (10.1)	3 (18.8)	11 (9.4)	0.376^e^
Adrenal failure	3 (2.2)	1 (7.1)	2 (1.7)	0.288^e^
Severe renal failure	19 (38.0)	2 (40.0)	17 (38.6)	NA
Hepatic failure	7 (4.1)	0 (0.0)	7 (4.7)	> 0.999^e^
Cachexia/significant malnourishment	19 (10.9)	3 (17.6)	12 (7.9)	0.181^e^
Brittle diabetes	21 (14.9)	6 (46.2)	13 (10.5)	**0.003**^**e**^
Weaning of corticosteroid drugs	10 (5.8)	1 (5.9)	9 (6.0)	> 0.999^e^
Previous hypo in the last 12 months	58 (47.5)	12 (85.7)	42 (40.4)	**0.001**^**a**^
Lifestyle factors				
Irregular meals	52 (37.1)	8 (50.0)	41 (34.7)	0.235^a^
Shift work	4 (2.4)	3 (18.8)	1 (0.7)	**0.003**^**e**^
Other	7 (4.0)	1 (5.9)	5 (3.3)	0.478^e^
Polypharmacy	147 (85.0)	7 (41.2)	135 (90.0)	**< 0.001**^**e**^
**High risk for complications after the event**				
Coronary heart disease	32 (25.0)	2 (14.3)	29 (26.4)	0.514^e^
Cerebrovascular disease	31 (24.2)	1 (7.7)	30 (27.0)	0.182^e^
Heart failure	48 (28.9)	0 (0.0)	48 (33.6)	**0.004**^**a**^
Peripheral artery disease	25 (22.1)	1 (7.7)	24 (25.0)	0.291^e^
Osteoporosis	8 (7.6)	1 (6.7)	7 (8.1)	> 0.999^e^
Living alone	33 (18.8)	9 (50.0)	22 (14.5)	**0.001**^**e**^

^a^ Chi-square test; ^b^ Kruskal-Wallis test; ^c^ Monte Carlo approach; ^d^ ANOVA; ^e^ Fisher’s exact test; ^f^ Each patient can have more than one; Polyphramacy: > 5 distinct drugs, for one or more conditions

Among T1DM patients, higher statistical differences between groups were found for disease duration (median 22.0 vs 10.0 years), diabetes management by self (72.2% vs 38.2%), eye and neuropathy complications (81.3% vs 46.2%; 50.0% vs 20.9%), and hypoglycemia in the last 12 months (85.7% vs 40.4%).

For T2DM patients statistically significant differences were found for age (78.0 vs 49.5), BMI (26.3 vs 22.9 kg/m^2^), primary healthcare follow-up (68.4% vs 22.2%), atherosclerotic disease complication (51.7% vs 14.3%), and polypharmacy as risk factor (90.0% vs 41.2%).

## Discussion

In the HIPOS-WARD study we evaluated patients with diabetes and a hypoglycemic episode that caused a hospitalization, focusing on the clinical characterization.

The hypoglycemia/hyperglycemia ratio was 0.27 for all DM patients and 0.38 in T2DM patients. For each hypoglycemia episode, there were approximately 4 hyperglycemic emergencies for all DM patients, and 3 for T2DM patients. These results were not as expected, and might be explained due to the safer and more effective state of hypoglycemia drugs, which lead to its decreasing occurrence. Additionally, the burden of hypoglycemia in inpatients in Portugal appears to be relevant but lower than the burden of hyperglycemia both in T1DM and T2DM. As no longitudinal data is available it is unknow how the situation has evolved over recent years. Lipska et al. [5] described between 1999 and 2011 in the USA a steady increase of admissions due to hypoglycemia, eventually overtaking admissions due to hyperglycemia in the first decade of this century. However, this was before therapies with a lower potential of hypoglycemia (DPP4i, SGLT2i and GLP1 analogues) were introduced or widely used [18, 19]. It is unknown if wider use of these agents, as well as decreasing use of SU type drugs and growing awareness of the risk of hypoglycemia reversed the trend once more. More recent insulin formulations are also associated with lower rates of hypoglycemia in T1DM and in some settings for T2DM [20–22]. In Portugal the use of anti-hyperglycemic agents with a low risk of hypoglycemia—DPP4i in particular—has increased substantially since early in the twenty-first century representing 25% of all oral drugs in 2015, while SU’s dropped from 54% in 2000 to 20% in 2015 [3]. Furthermore, increased awareness of hypoglycemia conveyed by national and international guidelines [21, 23] as well as the national studies HIPOS-ER [13], on severe hypoglycemia and HIPOS-PHARMA [24], a pharmacy level evaluation of mild/moderate hypoglycemia, might have contributed to a blunting in the rates of hypoglycemia. Also, glycemic target achievement in the National Health System (NHS) primary care has improved between 2010 and 2015 [3, 12]. This, overall, might suggest the importance of anti-hyperglycemic agent with a lower risk of hypoglycemia in containing the risk of severe hypoglycemia.

HIPOS-WARD only focused on the most severe hypoglycemia occurrences that lead to hospitalization and identified its clinical and economic burden [25] but does not cover the entire scope of the hypoglycemia problem.

Unfortunately, the lack of longitudinal data in Portugal cannot exclude the possibility–although unlikely–that the rate of hypoglycemia is still increasing. Irrespective of the causes our data reinforces the importance of anti-hyperglycemic agent with a low risk of hypoglycemia either as a contributing factor to curb it’s putative increase or to limit a future increase.

HIPOS-WARD reinforces the relevance of these therapies: the occurrence of hypoglycemic episodes was higher in patients on an insulin-based therapy followed by those on a secretagogue-based therapy, which are indeed associated to additional side effects and an increased risk of hypoglycemia [26–28]. Insulin+secretagogue therapy accounted for 10.2% of the sample and 9.7% were on non-secretagogue regimen. Therapeutic regimens based on or including secretagogues were more common in elderly patients and those followed in primary healthcare, which might be associated to treatment cost restrictions or related to clinical inertia. Drug prescription is indifferently managed by specialists/GPs and new antidiabetic drugs can be freely prescribed by all physicians. Although these patients are mostly seen at a primary care setting they are usually evaluated by in-hospital consultation when they fail to achieve control and need to add injectable therapy—like insulin—to their care or when they show complications from the disease. Our findings have shown that the insulin+secretagogue based therapy group was statistically associated with risk factors including disease duration and age, having higher proportion of patients with coronary heart disease, peripheral artery disease and osteoporosis. Also, a large group of patients reported nephropathy, a conventional risk factor for hypoglycemia in people with diabetes, due to multiple factors as the decrease of insulin clearance and it’s degradation in peripheral tissues, reduction in glycogen stores, reduced renal gluconeogenesis [29–31]. Also, commonly used antidiabetic drugs are renally excreted and have a prolonged half-life in patients with CKD, predisposing them to episodes of hypoglycemia. The confluence of these factors may contribute to a greater risk for hypoglycemia among patients with CKD and may be an unintended consequence of therapy to treat hyperglycemia in these patients [32]. Patients on secretagogue-based therapy had a higher proportion of cerebrovascular disease and heart failure. Again, this denotes the frailty of patients at higher risk of hypoglycemia and the importance of adopting strategies and therapies that minimize its risk [33, 34]. The actual increasing number of new AHAs with an intrinsic low risk of hypoglycemia are an important therapeutic option, however, patients and caregivers education are also crucial [35, 36].

This study also included T1DM patients, representing the first Portuguese national dataset on this population. Patients with T1DM, due to intensive insulin therapy, are usually associated with a higher risk of hypoglycemia [10] and in our study 85.7% of T1DM vs only 40.4% of T2DM had a hypoglycemic episode in previous 12 months. However, during the study period only 10.2% of the total hypoglycemia admissions were in T1DM and so hypoglycemia in T2DM represented the biggest burden of hypoglycemia events requiring hospitalization, highlighting that hypoglycemia is a very relevant issue in T2DM. It is well known that hypoglycemia triggered by secretagogues can be long-lasting, more severe and recurrent. Although expected, this data also shows that T1DM have a higher burden of microvascular complications whereas T2DM more atherosclerotic complications. This data alert to the importance of a patient centered approach to choosing appropriate pharmacologic treatment of blood glucose, including important comorbidities such as atherosclerotic cardiovascular disease. The rates of microvascular complications in T2DM ranged between 20.9–46.2% underscoring the importance of glycemic control on both groups in order to prevent these complications with great impact in quality of life [37–39].

Elderly, functionally dependent individuals (frail/non-frail) are exposed [40] and identified to have higher risk of hypoglycemia [10, 16, 33, 41–44]. Therefore, these patients should be cared for in a different way to mitigate the risk of this and other adverse events. Our results are consistent with a ward admitted patient population having T2DM, predominantly elderly, frail, with multiple comorbidities and moderately dependent, mainly treated with insulin and secretagogues. These hospitalizations were frequently preceded by similar and recent episodes, which reinforces the idea that these patients usually present recurrent hypoglycemia episodes, increasing the risk to develop complications at each occurrence. Moreover, more than half of patients need help (total/partial) to manage their diabetes treatment, denoting the frailty of these individuals at high risk of inadequate use of drugs and consequently at risk of having more severe episodes related to uncontrolled glycemia. The main risk factors for a hypoglycemia event were polypharmacy (> 5 distinct drugs, for one or more conditions) [45, 46] and age > 75 years, and heart failure was the most frequent risk factor for complications after an event.

Results are consistent with HIPOS-ER study [13], which triggered HIPOS-WARD by showing that, in a setting of ER, more than 40% of T2DM patients with severe hypoglycemia had been hospitalized as consequence of the event.

Overall, our data suggests that while hyperglycemia is still the major cause of hospital admissions for both types of diabetes, over 60% of all hypoglycemia admissions happened in patients with functional impairment and, in T2DM, elderly patients. This underscores that there is still work to be done in terms of avoiding clinical inertia and recurrence of hypoglycemic events in this patient group, questioning the use of secretagogue-based therapy and some combinations like SU + insulin, reinforcing the importance to increase the number of anti-hyperglycemic agents with low risk of hypoglycemia as a therapeutic option [35, 36] and developing educational and/or support programs for patients/caregivers, especially designed for populations at higher risk, in order to minimize the risk for complications.

Overall, the most likely causes of hypoglycemia were due to carbohydrate deficit, by a cause secondary to acute illness or related to insulin therapy. These potential causative factors should be addressed by systematically discussing it with all patients who are at increased risk of hypoglycemia as well as with their caregivers. For three quarters of patients hypoglycemia was the main driver for ward admission and the most frequent complications as result of the hypoglycemia episode that triggered the admission were neurologic and infection. This might indicate the Portuguese NHS approach by both specialists and primary care providers might have curbed the rates of hypoglycemia despite improving in diabetes target achievement. The growing use of new antihyperglycemic drug classes which have an intrinsic lower risk of hypoglycemia might be contributing to a decrease in the prevalence of severe hypoglycemia.

This study was not randomized or controlled, therefore results are primarily descriptive. Its observational nature may be considered a limitation and since no individual drug level information has been collected (only anti-hyperglycemic agent therapy group as pre-determined), it was not possible to determine specific causal associations. In order to minimize selection bias, patients were enrolled in a consecutive manner, in different centers throughout the country, which gives a wider overview of the information collected [41, 42]. However, there was clearly a higher number of T2DM patients and a low number of patients on non-secretagogue and insulin+secretagogue therapies in this study.

As a strength of the study we point out the specific tools (study questionnaire and literacy questionnaire) designed to adequately capture the information in accordance with its cross-sectional nature [44, 47]. Also, this study was part of an integrated series designed to evaluate adult patients with diabetes in pharmacies–HIPOS-PHARMA [24], in the emergency room–HIPOS-ER [13, 14], and hospitalized–HIPOS-WARD, contributing to a full country perspective.

In conclusion, in this study hyperglycemic emergencies were more frequent while the lack of longitudinal data doesn’t allow for trend projection. Elderly T2DM patients with functional impairment represent the bulk of admissions for hypoglycemia, highlighting there is still the need to optimize therapy in terms of the potential for hypoglycemia in this patient group, balancing glycemic target achievement with hypoglycemia avoidance. The study confirms higher rates of hypoglycemia in T1DM, highlighting the critical problem this complication represents, the overwhelming contribution of T2DM to the total burden, and the importance of this complication for both patients and the National Health System. Future efforts should also be taken to better characterize and prevent hyperglycemic occurrences especially in T2DM patients.

## Data Availability

Merck Sharp & Dohme Corp., a subsidiary of Merck & Co., Inc., Kenilworth, NJ, USA’s data sharing policy, including restrictions, is available at http://engagezone.msd.com/ds_documentation.php. Requests for access to the clinical study data can be submitted through the EngageZone site or via email to dataaccess@merck.com.
